# Assessment of Tumorigenic Potential in Mesenchymal-Stem/Stromal-Cell-Derived Small Extracellular Vesicles (MSC-sEV)

**DOI:** 10.3390/ph14040345

**Published:** 2021-04-09

**Authors:** Thong Teck Tan, Ruenn Chai Lai, Jayanthi Padmanabhan, Wei Kian Sim, Andre Boon Hwa Choo, Sai Kiang Lim

**Affiliations:** 1 Institute of Molecular and Cellular Biology, A*STAR, 8A Biomedical Grove, Singapore 138648, Singapore; tan_thong_teck@imcb.a-star.edu.sg (T.T.T.); lai_ruenn_chai@imcb.a-star.edu.sg (R.C.L.); eugene_sim@imcb.a-star.edu.sg (W.K.S.); 2 Bioprocessing Technology Institute, A*STAR, 20 Biopolis Way, Singapore 138668, Singapore; jayanthi_padmanabhan@bti.a-star.edu.sg (J.P.); andre_choo@bti.a-star.edu.sg (A.B.H.C.); 3 Department of Biomedical Engineering, Faculty of Engineering, National University of Singapore (NUS), 9 Engineering Drive 1, Singapore 117575, Singapore; 4 Department of Surgery, YLL School of Medicine, National University of Singapore (NUS), 5 Lower Kent Ridge Road, Singapore 119074, Singapore

**Keywords:** mesenchymal stem/stromal cell, small extracellullar vesicles, tumorigenicity

## Abstract

Mesenchymal-stem/stromal-cell-derived small extracellular vesicles (MSC-sEV) have been shown to ameliorate many diseases in preclinical studies. However, translating MSC-sEV into clinical use requires the development of scalable manufacturing processes for highly reproducible preparations of safe and potent MSC-sEVs. A major source of variability in MSC-sEV preparations is EV producer cells. To circumvent variability in producer cells, clonal immortalized MSC lines as EV producer lines are increasingly being used for sEV production. The use of sEVs from immortalized producer cells inevitably raises safety concerns regarding the tumorigenicity or tumor promoting potential of the EV products. In this study, cells from E1-MYC line, a MSC cell line immortalized with the *MYC* gene, were injected subcutaneously into athymic nude mice. At 84 days post-injection, no tumor formation was observed at the injection site, lungs, or lymph nodes. E1-MYC cells pre-and post-sEV production did not exhibit anchorage-independent growth in soft agar. Daily intraperitoneal injections of 1 or 5 μg sEVs from E1-MYC into athymic nude mice with FaDu human head and neck cancer xenografts for 28 days did not promote or inhibit tumor growth relative to the xenograft treated with vehicle control. Therefore, *MYC*-immortalized MSCs are not tumorigenic and sEVs from these MSCs do not promote tumor growth.

## 1. Introduction

Mesenchymal stem/stromal cells (MSCs) are multipotent cells that have the ability to differentiate into cells of the mesochyme lineage such as adipocytes, chondrocytes, and osteocytes. They can be easily isolated from adult tissues and extensively expanded ex vivo. They can differentiate into several cell types such as adipocytes, chondrocytes, and osteocytes. MSCs are currently the most clinically trialed cell type, with >1200 trials targeting many disease indications (https://clinicaltrials.gov/, accessed on 1 February 2021). The mechanism by which MSC exert their therapeutic effects has evolved over the years. Initially, it was hypothesized that transplanted MSCs home to the site of injury where they engraft and differentiate into the relevant cell types to replace dead or damaged cells. However, it was often observed that despite functional improvements, MSCs or differentiated MSCs were not detected in biologically significant numbers in affected tissues. In 2006, Caplan and Dennis [1] postulated that as MSCs secrete many bioactive molecular species [2], MSCs could exert their therapeutic activity through these secreted molecules rather than by direct cellular interactions. In 2008, it was observed that the cardioprotective agent in MSC secretion was enriched in a fraction of MSC-conditioned media with molecular weights (MW) larger than 1000 kDa [3]. This fraction was subsequently found to be rich in 110 and 130 nm small extracellular vesicles (sEVs), which were termed exosomes at that time [4]. In a similar study, a fraction of MSC conditioned media that was highly enriched for EVs (80 nm to 1 μm) termed microvesicles protected against acute renal tubular injury in a mouse model [5]. As the size range of the reported MSC exosome preparation is a subset of the reported microvesicle preparation, it is possible that both preparations exert their therapeutic effects through similar EV types. Subsequently, head-to-head comparison studies demonstrated that EV preparations were as therapeutically effective as their producer cells in different pre-clinical models [6,7,8]. Today, it is widely accepted that the therapeutic effects of MSCs are mediated significantly by sEVs with diameters between 50 and 200 nm [6].

EVs are bi-lipid membrane vesicles that are released into the extracellular space by virtually all cell types. Several EV types have been described to date such as exosomes, microvesicles, ectosomes, membrane particles, exosome-like particles, and apoptotic bodies [7]. The most widely used methods for EV isolation are based on biophysical properties such as size or density. As there are significant overlaps in these properties among the different EV types, most EV preparations are heterogeneous, including MSC-sEV preparations that are enriched in 50–200 nm EVs. MSC-sEVs like most EVs have a characteristic composition of membrane lipids, proteins, and RNAs, and function primarily as intercellular communication vehicles to transfer bioactive proteins, lipids, and nucleic acids between cells to elicit biological responses in recipient cells (as reviewed [8]). We have previously reported that our MSC-sEV preparations contain at least three distinct EV populations, including exosomes derived from the endosomes [9,10]. Therefore, we will use the term ‘’MSC exosomes’’ to refer to our MSC-sEV preparations.

MSC-sEVs have been shown to be efficacious in pre-clinical animal models of human diseases, and many efforts have been made to translate MSC-sEVs into therapeutic products. Several academic societies, namely, the International Society for Cell and Gene Therapy (ISCT), International Society for Extracellular Vesicles (ISEV), and the Society for Clinical Research and Translation of Extracellular Vesicles Singapore (SOCRATES) have organized workshops and written papers to facilitate such efforts [6,11] (Gimona et al., in press). A major consensus on the challenges in manufacturing MSC-sEV preparations of robust reproducible identity and potency is the sEV producer cell source especially when primary cells are used. Primary cells have finite lifespan and will require frequent replenishment. As there is significant variability between donors and also within donors due to changes in age and health status, cell replenishment will introduce variability in the quality of the producer cells. To mitigate this challenge, we have proposed generating immortalized clonal cell lines with infinite expansion potential [12].

We previously reported the transformation of human ESC-derived MSCs using the *MYC* gene to immortalize the MSCs and generate a clonal cell line, E1-MYC [12]. E1-MYC cells grow faster and have increased telomerase activity while retaining the parental karyotype. The exosomes were highly similar before and after immortalization. Biochemical assay, mass spectrometry analysis, western blot hybridization, and antibody arrays revealed the presence of membrane lipids, namely, cholesterol, sphingomyelin, and phosphatidylcholine, and a proteome of ~1000 proteins including exosome-associated proteins such as CD81, CD9, and ALIX [4,13,14] Like most EVs, they also have a diverse RNA cargo as determined by array hybridization, reverse transcription polymerase chain reaction (RT-PCR), and RNA sequencing [9,15]. The presence of exosomes, i.e., vesicular particles of ~100–200 nm with membrane CD81, was also confirmed by transmission electron microscopy (TEM) and immunoelectron microscopy [9,16].

The use of MSC exosomes from immortalized MSC producer cells inevitably raises safety concerns regarding the tumorigenicity or tumor promoting potential. MSCs generally have a strong safety record, as evidenced by the large number of clinical trials using MSCs, and it is reasonable to assume that non-living secretions from MSCs such as EVs would be as safe if not safer. However, immortalization of MSCs with a proto-oncogene such as MYC may alter the safety profile of the MSCs and their secretion and confer tumorigenic or tumor promoting activities on the cells or their secretion.

In this study, we investigated the tumorigenicity of E1-MYC cells, and the effect of E1-MYC-derived exosomes on tumor growth. We tested E1-MYC cells for anchorage-independent growth in soft agar and tumor formation into athymic nude mice. We also tested the effect of E1-MYC-derived exosomes on tumor progression in an athymic nude mouse model of Head and Neck Cancer xenografts.

## 2. Results

### 2.1. E1-MYC Does Not Form Tumors In Vivo

To determine tumorigenicity of E1-MYC, 1 × 10^7^ cells were subcutaneously injected into the right lateral thorax of athymic nude mice. Cells from HT-1080, a fibrosarcoma line, and MRC-5, a fibroblastic line, were used as positive and negative controls, respectively. The mice in the positive control group (HT-1080) were submitted for necropsy on days 7, 11, and 19 post cell injection. On day 21 post cell injection, half of the mice from E1-MYC group and MRC-5 negative control group were submitted for necropsy. The remaining mice were submitted for necropsy on day 84 post cell injection all mice injected with HT-1080 cells developed neoplastic masses at the injection site. One mouse had masses in the lungs, another had masses in the right axillary lymph node, and four had additional smaller masses next to the primary mass. Microscopic examinations of the masses revealed a composition of neoplastic spindloid cells consistent with fibrosarcoma (Table 1). None of the mice injected with E1-MYC and MRC-5 cells showed any lesion at the injection site or in other off-site tissues (Table 1). These results indicate that E1-MYC cells are not tumorigenic.

### 2.2. Anchorage-Independent Growth Assay

**Figure 1:**
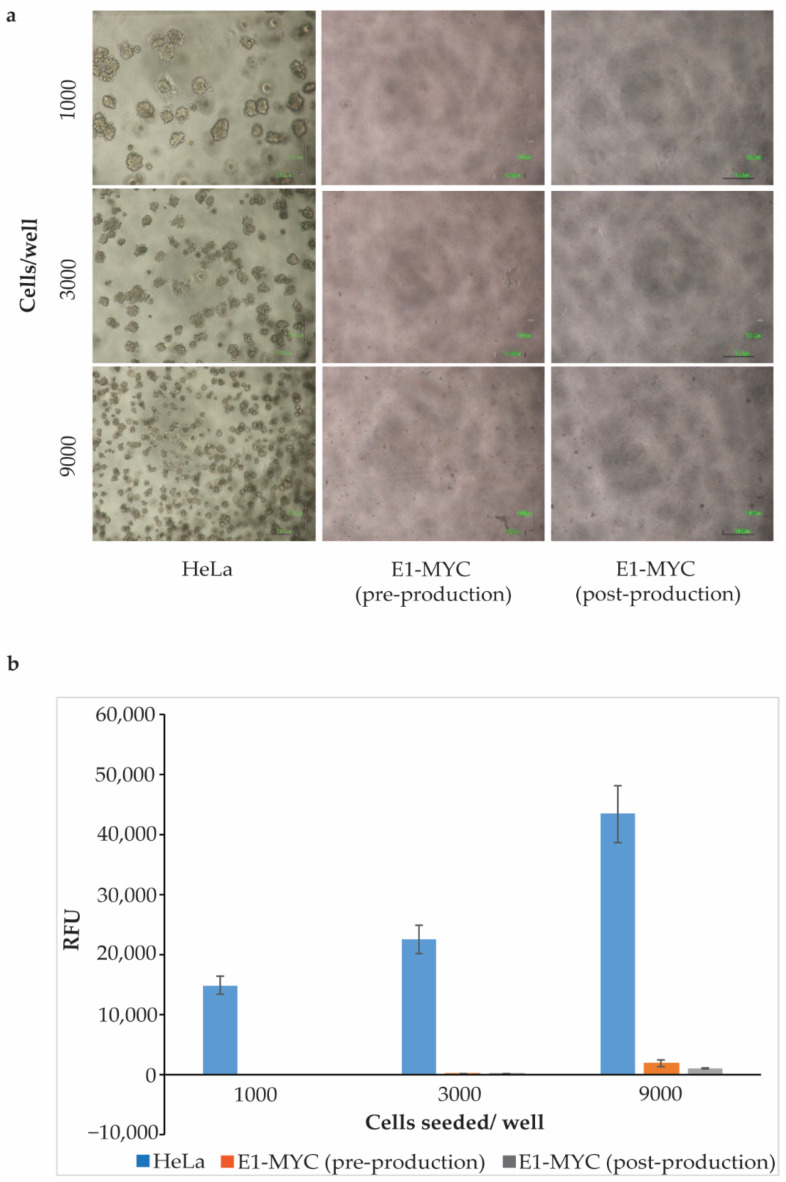
Anchorage-independent growth. HeLa cells and E1-MYC cells from pre- or post-production medium were seeded at three different densities and cultured for 7 days. (**a**) Representative images (4× magnification) HeLa and E1-MYC cultures after 7 days. (**b**) Relative cell growth as determined by DNA-binding dye fluorescence after 7 days in culture. Results represent the mean (*n* = 3, triplicate for each sample) ± SD.

To evaluate if E1-MYC cells were modified after being used for exosome production, we tested E1-MYC cells pre- and post-exosome production for anchorage-independent growth assay. For MSC exosome production, E1-MYC cells were first expanded in a serum-containing growth medium and then in serum-free chemically defined medium for exosome production. At the end of expansion just before being transferred to serum-free defined medium for exosome production, i.e., pre-production, and after conditioning the defined medium for exosome production, i.e., post-production, cells were harvested and tested for anchorage-independent growth using the CytoSelect 96-well Cell Transformation Assay (#CBA-130-T, Cell Biolabs Inc., San Diego, CA, USA) with HeLa cells as a positive control. Three different cell densities—1000, 3000, and 9000 cells/well—were seeded in triplicates. After 7 days in culture, HeLa cells showed robust colonies formation but no colony was observed in E1-MYC from either pre- or post-production phases (Figure 1). Relative cell concentration in the cultures was determined using a DNA-binding fluorescent dye assay kit, Cyquant quantification. Unlike HeLa cells, E1-MYC cells pre- and post-exosome production had minimal anchorage-independent growth.

### 2.3. The Effects of MSC-sEV on Tumor Progression

We previously reported that the MYC protein was undetectable in E1-MYC cell secretion and exosomes and that the exosomes were similar before and after MYC transformation, demonstrating that MSC exosomes do not carry MYC oncoprotein to promote tumor growth [12]. As shown above, E1-MYC cells do not form tumors. However, it is possible that *MYC* transformation may confer tumor-promoting activity on the exosomes. Here, we test this possibility on a mouse model of FaDu head and neck cancer xenograft. FaDu cells form low-grade carcinoma (grade I) in nude mice [17] and are therefore well suited to detect possible pro-tumorigenic activity of MSC exosomes. Furthermore, the cells could be grafted subcutaneously to form epidermoid carcinoma. This greatly facilitated continuous measurement and monitoring without the need to sacrifice the mice. After establishment of the xenografts, the mice were injected intraperitoneally daily with vehicle, one or five µg (protein weight) of exosomes for 28 days or 4 weeks (Table 2). Paclitaxel [18,19] (an anti-microtubule agent) was used as a benchmark reference for any anti-tumor activity. As such, the paclitaxel was injected IV twice a week as widely practiced for optimal anti-tumor activity, and not as per administration of exosomes.

**Figure 2:**
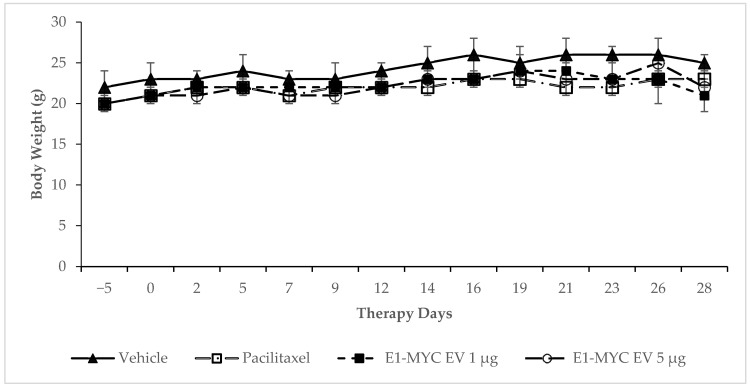
Effect of E1-MYC exosome on body weight. Mice were injected with either vehicle, Paclitaxel, or two different doses of E1-MYC exosome (1 µg or 5 µg), and their body weight was recorded thrice weekly. No statistically significant differences in body weight between the different treatment groups were observed. Results represent the mean ± SD, *n* = 8 for all groups from day 0 to day 16. Mice with tumor volume > 2000 mm^3^ were euthanized and caused the *n* to gradually drop to 2 for the vehicle and exosome groups at day 28.

To monitor the overall well-being of the mice, body weight of the mice was measured. There was no statistically significant difference among the body weights of mice receiving vehicle, Paclitaxel, or E1-MYC exosomes (both 1 µg and 5 µg doses) (Figure 2), suggesting that MSC exosomes from E1-MYC cells did not have any adverse effect on the animals.

**Figure 3:**
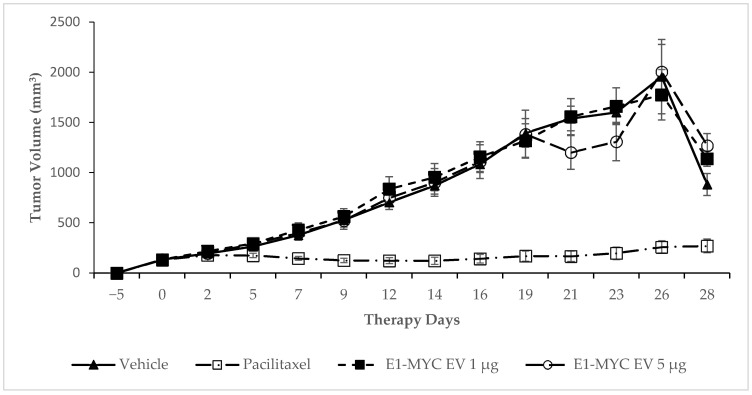
Effect of E1-MYC exosome on tumor growth. Mice were injected with either vehicle, Paclitaxel, or two different doses of E1-MYC exosome (1 µg or 5 µg), and tumor volume was recorded thrice weekly. Animals treated with E1-MYC exosome had no difference from vehicle control in terms of tumor growth. Results represent the mean ± SEM, *n* = 8 for all groups from day 0 to day 16. After day 16, tumor volume in some exosome- or vehicle-treated mice started to exceed 2000 mm^3^. Euthanizing mice with large tumor volumes reduced the average increase in tumor volume and eventually reduced the tumor volume. At day 28, the vehicle and exosome groups each had only two surviving mice.

To examine the effect of E1-MYC exosomes on tumor growth, tumor volume in each mouse was determined using the formula: tumor vol = length × width × width × ½. Tumor volume in mice receiving either 1 µg or 5 µg E1-MYC exosomes was not statistically different from those in vehicle- treated mice (Figure 3). On the other hand, mice treated with Paclitaxel had significantly lower tumor volume than mice treated with vehicle from day 5 onwards. At the end of 28 days, the number of surviving mice was 2 for the vehicle group and each of exosome groups. The Paclitaxel group had 8 surviving mice.

## 3. Discussion

The therapeutic efficacy of MSC-exosomes/sEVs in animal models has generated much optimism about its clinical applications. As with all therapeutics, a primary issue of concern is the safety of the product such as the potential for tumorigenic activity.

MSC transplantations are generally considered safe, as evidenced by the numerous phase 1 safety clinical trials completed to date. Several studies have shown that primary MSC does not form tumors [20,21,22,23,24,25]. Hence, it is generally assumed that MSC exosomes or sEVs are as safe if not safer. However, the use of primary cells cannot sustain large-scale manufacturing of MSC exosomes with consistent reproducible identity and potency. To mitigate this challenge, we have transformed MSCs with a proto-oncogene, MYC to derive a clonal immortalized cell line [12]. The transformed cells retained the karyotype of the parental cells and produced exosomes with similar characteristics and therapeutic potency as the untransformed cells. Although MYC protein was not detected in the secretion or exosomes, the tumorigenic potential of the transformed cells or their exosome has not been directly addressed.

In this study, we specifically demonstrated that cells from the transformed MSC cell line, E1-MYC, do not form tumors in nude mice and that the cells did not exhibit anchorage-independent growth at pre-or post-exosome production. These observations are consistent with previous study reports that *h-TERT* immortalized MSC lines do not induce tumor formation [26,27,28].

As MSC-EVs are non-living, so they cannot form tumors. However, it is possible that they can affect tumor growth. The effects of MSC-EVs on tumor progression are somewhat controversial. Some studies reported that MSC-EVs inhibited the proliferation of tumors [29,30,31], while others reported that MSC-EV contributed to tumor growth and metastasis [32,33]. All the MSC-EVs used in these studies were produced by naïve MSCs. In contrast to these studies, MSC exosomes used here were produced by a MYC-transformed MSC, E1-MYC cells. Despite this, these MSC exosomes also did not inhibit or promote tumor growth. The conflicting results on the tumorigenicity of MSC-sEVs could be due to heterogeneity of MSC source, different methodology of EV isolation, or different tumor models used.

In conclusion, our data demonstrate that transformation of MSCs by *MYC*, a proto-oncogene, to generate immortalized cells did not confer tumorigenic activity on the cells. Our method for exosome production did not alter the growth of the cells such that they acquired anchorage-independent growth or generate tumor-promoting MSC exosome preparations. Therefore, immortalization of MSCs for exosome production is a viable option for large-scale production of safe exosome preparations for therapeutic use.

## 4. Materials and Methods

### 4.1. Culture of MSC and Preparation of MSC-Exosome

Immortalized E1-MYC 16.3 human ESC-derived mesenchymal stem cells were cultured in DMEM with 10% fetal calf serum as previously described [12]. To prepare MSC exosomes, 80% confluent cell culture was grown in a chemically defined medium for three days to generate a conditioned medium (CM) as previously described [4,34,35]. The defined medium was prepared as follows: 480 mL DMEM (Thermo Fisher, #31053), 5 mL NEAA (Thermo Fisher, #11140-050), 5 mL L Glutamine (Thermo Fisher, #25030-081), 5 mL Sodium Pyruvate (Thermo Fisher, #11360), 5 mL ITS-X (Thermo Fisher, #51500-056), and 0.5 mL 2-ME (Thermo Fisher, #21985-02). This was supplemented with 0.1 mL bFGF (0.5 ng/μL 0.2%BSA in PBS (+) and 0.005 mL PDGF (100 ng/μL PBS (+)). These latter components were obtained as follows: Bovine Serum Albumin or BSA (Sigma-Aldrich, #A9647), PDGF (AB CYTOLAB, #100-00), bFGF (Thermo Fisher, #13256-029), and PBS (+) (Thermo Fisher, #14040-133). The CM was size fractionated and concentrated 50× by tangential flow filtration using a membrane with a molecular weight cut-off (MWCO) of 100 kDa (Sartorius, #VS15T41) to generate the MSC exosome preparation. The MSC exosome preparation was assayed for protein concentration using a Coomassie Plus (Bradford) Assay Kit (ThermoFisher Scientific, #23236), and the exosome preparations were quantified by the protein concentration. All batches of exosome used in this study were determined by Nanoparticle tracking analysis on a ZetaView instrument (Particle Matrix GmbH, Germany) to have 1.46 × 10^11^ ± 2.43 × 10^10^ particles per ug protein and particle modal size of 138.62 ± 4.45 nm using the parameters (sensitivity = 90, shutter = 70, frame rate = 30, min brightness = 25, min area = 5, max area = 1000). Each batch of MSC exosome preparation was also confirmed by western or ELISA to have CD81 and CD73. The exosome preparations were filtered with a 0.22 μm filter (Merck Millipore, Billerica, MA, USA) and stored in −20 °C freezer until they were used in 4.2. in vitro tumorigenicity assay.

### 4.2. In Vitro Tumorigenicity Assay

To evaluate tumorigenicity of E1-MYC cells pre and post exosome production, anchorage-independent growth was assessed with the soft agar colony formation assay using the CytoSelect^TM^ 96-well Cell Transformation Assay (#CBA-130-T, Cell Biolabs Inc., San Diego, CA, USA). Briefly, a 0.6% base agar layer containing DMEM and FBS was prepared in a 96-well plate. E1-MYC cells from expansion and production phases, and HeLa cells, were suspended in DMEM containing FBS and 0.4% agar solution and plated onto the base layer. Three different cell densities were plated in triplicates, 1000, 3000, and 9000 cells/well. Cells were incubated at 37 °C with 5% CO_2_ for 7 days before quantitation with a CyQuant^®^ GR Dye assay kit (#1010-T, Cell Biolabs Inc., San Diego, CA, USA).

### 4.3. Cell Implantation and Histological Evaluation for Tumor Formation

In vivo tumorigenicity assay and histological evaluation were performed by Charles River Laboratories (Protocol code: PR-4-10). Briefly, three groups of ten athymic nude mice were subcutaneously injected with 1 × 10^7^ of either E1-MYC, a positive control fibrosarcoma cell line HT-1080, or a negative control fibroblast cell line MRC-5. Animals in the positive control group (HT-1080) were submitted for necropsy on days 7, 11, and 19 post cell injection. On day 21 post cell injection, half of the mice from E1-MYC group and MRC-5 negative control group were submitted for necropsy. The remaining mice were submitted for necropsy on day 84 post cell injection. To examine the tumorigenic and biodistribution potential of the cells injected, tissues from the injection site, right axillary lymph lode, lungs, liver, spleen, kidneys, and any general gross lesions were harvested. The harvested tissues were fixed in formalin, embedded in paraffin, and sectioned at five microns, mounted on a glass slide and stained with hematoxylin and eosin.

### 4.4. Pro-Tumorigenic Activity of MSC-sEV

Pro-tumorigenic activity of MSC-sEV in a Fadu xenograft model in athymic nude mice for human head and neck carcinoma were done by a CRO, Washington Biotechnology Inc (Protocol code: 17-074.4). Four groups of 8 mice were injected with 5 × 10^6^ Fadu cells subcutaneously into the right flank of each mouse. Upon development of tumors of sufficient size, the dosing regimen (Table 2) was initiated. The animal weights and tumor measurements were recorded three times a week. The xenograft tumors were measured with a digital caliper. Tumor volume was calculated using the formula: tumor vol = length × width × width × ½. During the study, mice with oversize tumor (>2000 mm^3^) were euthanized and removed from the study. Tumor sizes were analyzed using Student’s *t*-test. *p*-values < 0.05 were considered as statistically significant.

## Figures and Tables

**Table 1:** In vivo tumorigenic assay. Pathological observations after implantation of E1-MYC, HT-1080, and MRC-5 in athymic nude mice.

Parameter	E1-MYC	HT-1080	MRC-5
No. of animals	10	10	10
Lesion observed			
-injection site	0	10	0
-axillary lymph	0	1	0
-lungs	0	1	0
-spleen	0	0	0
-liver	0	0	0
-kidney	0	0	0
-additional site	0	4	0

**Table 2:** Treatment regime of tumor model. Group treatments of animals following development of tumor.

Group	No. Mice	Test Material	Dose (mg/kg)	ROA	Frequency
1	8	Vehicle	200 µL/per mouse	IP	Daily for 4 weeks
2	8	Paclitaxel	15 mg/kg	IV	Twice per week
3	8	E1-MYC exosomes	1 µg/per dose	IP	Daily for 4 weeks
4	8	E1-MYC exosomes	5 µg/per dose	IP	Daily for 4 weeks

## Data Availability

Data is contained within the article.
